# Supplementary material for: The Systems Biology Research Tool: evolvable open-source software
Source: BMC Syst Biol. 2008 Jun 29;2:55. doi: 10.1186/1752-0509-2-55 (PMC2446383; doi:10.1186/1752-0509-2-55)
Supplement: Additional file 1 — SBRT Archive. An archive of the current version of the Systems Biology Research Tool. [file 1752-0509-2-55-S1.zip › sbrt-1.4.0/doc/developers_guide/api/index-files/index-4.html]

D-Index


|  |  |  |
| --- | --- | --- |
| **Overview** \| Package \| Class \| Use \| **Tree** \| **Deprecated** \| **Index** \| **Help** | |  |
| **PREV LETTER**   **NEXT LETTER** | **FRAMES**    **NO FRAMES**     **All Classes** |  |


A B C D E F G H I J K L M N O P Q R S T U V W X Y Z 

---


## **D**

**DASH** - Static variable in class sbrt.shell.text.SpecialChars: - **DATA\_HEADERS** - Static variable in class sbrt.shell.mng.AbstractProcessManager: The keyword used to denote the data headers of, say, an output file. **debugPrintln(Object)** - Static method in class sbrt.kernel.util.Util: Prints the provided object to `System.err`, preceded by a carriage return character. **DEFAULT\_CONST\_TOL** - Static variable in class sbrt.kernel.fba.comp.FbaOptComputer: The default constraint tolerance. **DEFAULT\_CONSTRAINT\_TOLERANCE** - Static variable in class sbrt.shell.mng.fba.FbaManager: The default constraint tolerance. **DEFAULT\_CONSTRAINT\_TOLERANCE** - Static variable in class sbrt.shell.mng.geom.CdHarManager: The default constraint tolerance. **DEFAULT\_MIN\_CHORD\_LENGTH** - Static variable in class sbrt.shell.mng.geom.CdHarManager: The default constraint tolerance. **DEFAULT\_P** - Static variable in class sbrt.shell.mng.fba.InitialPointManager: The default P intercept value. **DEFAULT\_SAFETY\_LEVEL** - Static variable in class sbrt.kernel.fba.comp.FbaOptComputer: The default saftey level. **DEFAULT\_SAFETY\_LEVEL** - Static variable in class sbrt.shell.mng.fba.FbaOptManager: The default safety level used by `FbaOptManager.getSafetyLevel(ManagerInput, PrintWriter)`. **DEFAULT\_TOLERANCE** - Static variable in class sbrt.kernel.fba.comp.CdHarFluxVectorComputer: The default constraint tolerance. **delete(Set<String>)** - Method in class sbrt.kernel.fba.comp.RxnDeletionComputer: Computes and returns the results of deleting the reactions whose names appear in the provided set. **DEV\_NULL** - Static variable in class sbrt.shell.mng.AbstractProcessManager: A print writer that discards all data it receives. **DevNull** - Class in sbrt.shell.io: This class is used to mimic /dev/null from Unix-like operating systems. **DevNull()** - Constructor for class sbrt.shell.io.DevNull: Constructs new "/dev/null" object. **DimensionException** - Exception in sbrt.kernel.math.geom: This class is used to indicate invalid dimensions. **DimensionException(int)** - Constructor for exception sbrt.kernel.math.geom.DimensionException: Constructs a new dimension exception. **dimensions()** - Method in interface sbrt.kernel.math.geom.ConvexPolytope: Returns the dimensions of the space in which this polytope lies. **dimensions()** - Method in class sbrt.kernel.math.geom.HashConvexPolytope: Returns the dimensions of the space in which this polytope lies. **dimensions()** - Method in class sbrt.kernel.math.geom.HashConvexPolytopeOld: Returns the dimensions of the space in which this polytope lies. **DirectedGraph**<N> - Class in sbrt.kernel.math.graph\_theory: This class is used to represent directed graphs. **DirectedGraph(Set<DirectedGraph.Edge<N>>)** - Constructor for class sbrt.kernel.math.graph\_theory.DirectedGraph: Constructs a new directed graph from the provided collection of edges. **DirectedGraph.Edge**<N> - Class in sbrt.kernel.math.graph\_theory: This class is used to represent the unidirectional edges of a directed graph. **DirectedGraph.Edge(T, S)** - Constructor for class sbrt.kernel.math.graph\_theory.DirectedGraph.Edge: Constructs a new directed edge from the provided source and sink nodes. **DirEdgeFormat**<N> - Interface in sbrt.shell.text.math: This interface is used to represent formats for the edges of directed graphs. **DirEdgeFormatV1**<N> - Class in sbrt.shell.text.math: This class is a concrete implementation of `DirEdgeFormat`. **DirEdgeFormatV1(SimpleFormat<N>)** - Constructor for class sbrt.shell.text.math.DirEdgeFormatV1: Constructs a new directed edge format. **DiscreteProgressMonitor** - Class in sbrt.kernel.util: This class is used to monitor the progress of a process that occurs in discrete events. **DiscreteProgressMonitor()** - Constructor for class sbrt.kernel.util.DiscreteProgressMonitor: Constructs a new discrete progress monitor. **DOLLAR** - Static variable in class sbrt.shell.text.SpecialChars: $ **DOT** - Static variable in class sbrt.shell.text.SpecialChars: . **DoubleEquiv** - Class in sbrt.kernel.math: This class is used to compare double precision numbers for equivalence. **DoubleEquiv(double)** - Constructor for class sbrt.kernel.math.DoubleEquiv: Creates a new double precision number equivalence tester with the provided tolerance. **DoubleFormat** - Interface in sbrt.shell.text: This interface is used to represent a format for double precision numbers. **DoubleFormatV1** - Class in sbrt.shell.text: This class is a concrete implementation of `DoubleFormat`. **DoubleFormatV1()** - Constructor for class sbrt.shell.text.DoubleFormatV1: **DoubleFormatV2** - Class in sbrt.shell.text: This class is a concrete implementation of `DoubleFormat`. **DoubleFormatV2()** - Constructor for class sbrt.shell.text.DoubleFormatV2: Constructs a new double format for finite numbers. **DoubleMatrix**<R,C> - Interface in sbrt.kernel.math.algebra: This interface is used to represent matrices whose elements are strictly double precision numbers. **DoubleMatrixBuilder**<R,C> - Interface in sbrt.kernel.math.algebra: This interface is used to represent mutable matrices whose elements are strictly double precision numbers. **DoubleMatrixWrap**<R,C> - Class in sbrt.kernel.math.algebra: This class is used to wrap mutable matrices as an immutable `DoubleMatrix`. **DoubleMatrixWrap(DoubleMatrix<R, C>)** - Constructor for class sbrt.kernel.math.algebra.DoubleMatrixWrap: Constructs a new wrapper around the provided matrix. **DummyConsole** - Class in sbrt.ext.R: This class is used to make dummy consoles for the JRI. **DummyConsole()** - Constructor for class sbrt.ext.R.DummyConsole

---


|  |  |  |
| --- | --- | --- |
| **Overview** \| Package \| Class \| Use \| **Tree** \| **Deprecated** \| **Index** \| **Help** | |  |
| **PREV LETTER**   **NEXT LETTER** | **FRAMES**    **NO FRAMES**     **All Classes** |  |


A B C D E F G H I J K L M N O P Q R S T U V W X Y Z 

---
